# Acute kidney injury in patients undergoing elective primary lower limb arthroplasty

**DOI:** 10.1007/s00590-021-03024-x

**Published:** 2021-06-03

**Authors:** Luke Farrow, Stacey Smillie, Joseph Duncumb, Brian Chan, Karen Cranfield, George Ashcroft, Iain Stevenson

**Affiliations:** 1 Institute of Applied Health Sciences, University of Aberdeen, Aberdeen, UK; 2 Woodend Hospital, Aberdeen, UK; 3 University Hospital of Ayr, Ayr, UK; 4 Royal Infirmary of Edinburgh, Edinburgh, UK

**Keywords:** Acute kidney injury, Chronic kidney disease, Arthroplasty, Orthopaedics, Hip, Knee

## Abstract

**Purpose:**

Recent research has outlined the increasing incidence of acute kidney injury (AKI) and its effect on morbidity/mortality. There is evidence that current rates are significantly under-reported nationally, with uncertainty about pre-operative factors that might influence AKI reduction and the impact on other healthcare outcomes such as mortality and later Chronic Kidney Disease (CKD) development. We set out to help address these current deficiencies in the literature.

**Methods:**

A retrospective cohort study was undertaken using data collected from patients undergoing elective primary lower limb arthroplasty within our institution from 01/10/16–31/09/17 with a 2-year follow-up.

**Results:**

53/782 (6.8%) patients had an AKI during the study time period. This was associated with a longer inpatient stay (*p* < 0.001). There was no significant difference in 30-day mortality (*p* = 0.134), 30-day readmission (*p* = 1.00) or later CKD development (*p* = 0.63). Independent predictors of AKI were as follows: Diabetes (OR 2.49; 95%CI 1.15–5.38; *p* = 0.021), CKD (OR 4.59; 95%CI 2.37–8.92; *p* < 0.001) and Male sex (OR 2.61; 95%CI 1.42–4.78; *p* = 0.002).

**Conclusions:**

AKI in those undergoing hip and knee arthroplasty remains under-reported at a national level. AKI development was associated with an increased length of stay, but not long-term healthcare outcomes. This may be due to the mechanism of AKI development or the low absolute numbers of AKI suffered. We have identified three pre-operative factors (Diabetes, CKD & Male Sex) that were independently predictive of AKI. Targeted interventions may reduce the risk of AKI after lower limb arthroplasty.

## Introduction

Acute kidney injury (AKI) is a sudden deterioration in kidney function usually multifactorial in origin [1]. There is increasing evidence that even a transient AKI can have a subsequent impact on the risk of increased short- and long-term mortality, as well as increased hospital costs and length of stay [1–3].

Although there has previously been little interest in AKI in orthopaedic surgery, recent data from the Scottish Arthroplasty project report have suggested an exponential increase in the incidence of AKI in elective arthroplasty surgery from 0.3 to 0.5% in 2000 to 1.9-2.2% in 2019 [4]. Recent studies focussing on orthopaedic surgery have suggested that AKI is associated with an increased length of stay in elective surgery [5], as well as increased mortality [6] and costs [7].

Whilst other studies have examined potential risk factors for AKI in orthopaedic patients [7–9], there is limited evidence to guide targeted intervention strategies to reduce AKI risk. Identification of patients at high risk of AKI is particularly important in a financially constrained heath system such as the NHS, where the significant increased cost associated with AKI has negative implications for population health and economics.

There is evidence in other healthcare settings that the impact of AKI is not just related to short-term complications and outcomes, but also to the development of future Chronic Kidney Disease (CKD) [2, 10]. There has however been limited investigation about how the development of AKI following hip and knee arthroplasty might influence longer term CKD risk.

To help address the current deficiencies in the literature, we therefore set out to examine:

I. The rate of AKI within our institution in patients undergoing primary elective lower limb arthroplasty
II. Pre-operative patient factors that may influence AKI risk
III. Impact of AKI development on patient outcomes including the risk of CKD development at two years post-operatively.

## Methods and Materials

A retrospective cohort study was undertaken using data collection from all patients undergoing elective primary lower limb arthroplasty within a single large regional university hospital in Scotland from 01/10/16 to 31/09/17. Eligible patients were identified using an online theatre management system. Individuals were excluded if they were undergoing complex primary (such as that for development hip dysplasia & acetabular fracture) or revision surgery. Patients who had end-stage renal failure (ESRF) requiring renal replacement therapy (RRT) were also excluded.

Electronic patient records were used to collect relevant pre-operative patient and outcome variables (determined via prior analysis of relevant literature regarding AKI in Trauma & Orthopaedic surgery) including the following:

- **Demographic:** Age, Sex, Body Mass Index (BMI)
- **Pre-operative:** Serum Creatinine, Diuretic use, Non-Steroidal Anti-Inflammatory (NSAID) use, Angiotensin II Receptor Blocker (ARB) or Angiotensin Enzyme Inhibitor (ACEI) use, significant Cardiovascular Disease (CVD) [previous Stroke, Myocardial Infarction or Peripheral Vascular Disease (PVD)] and Diabetes (medication controlled)
- **Post-operative:** Post-operative serum creatinine, NSAID use, length of stay, 30-day mortality, 30-day readmission and risk of CKD development at a minimum 2-year post-operative follow-up.

AKI was classified according to the (AKI Network) AKIN criteria [11]; with a definition of an absolute risk in serum creatinine of > 26.4 micromole per litre or a 150% rise from baseline. Urine output was not utilised as part of the data collection due to concerns over the accuracy of recorded results. The highest post-operative creatinine value in the 5 days following surgery was used for the calculation of AKI.

Statistical analysis was performed using SPSS 24 for Windows. The rate of AKI was calculated using the number of individuals undergoing primary elective arthroplasty as the denominator. A Mann–Whitney U test was used to assess the effect of AKI development on length of stay. 30-day mortality and 30-day readmission were examined using Fisher’s exact test. Subsequent CKD development (or worsening of CKD status if a pre-operative diagnosis of CKD was present) was also evaluated at a minimum of 2-year post-operative follow-up, including comparison for those who suffered an AKI versus those who did not. CKD was defined as an eGFR < 60 persistently for 3 months or more. If a patient had only one test in the entire post-discharge period, this was also included as CKD development. In those patients suffering an AKI, bivariate logistic regression was used to determine the risk of AKI development for the pre-operative variables of Age, Sex, BMI, surgery type, CKD, ACEI/ARB, Diabetes, Diuretics, Pre-operative NSAIDs and CVD. Multivariate logistic regression was then performed with a *p* ≤ 0.10 significance level to determine which factors were independently predictive of AKI. Given there were only 53 patients that sustained an AKI the multivariate regression was limited to five variables to avoid overfitting the data. A chi-squared analysis was then performed using a dummy variable containing any of the independently predictive pre-operative variables.

The study was registered with the local clinical effectiveness team; formal ethics approval was not required due to the retrospective non-interventional nature of the study design as confirmed by the HRA decision tool. All data collection and storage were carried out in accordance with the Caldicott principles.

## Results

A total of 788 patients were eligible for inclusion in the analysis (61.9% female). A total of 782 patients had sufficient data to allow for assessment of AKI development, with 53% undergoing THR and 47% TKR. The mean age was 68 (SD 10.6).

In total, 53/782 (6.8%) patients undergoing elective primary lower limb arthroplasty had an AKI during the study time period. According to the AKIN criteria for AKI severity 45 of these were Type I AKI, 6 were Type II and 2 were Type III. No patients required renal replacement therapy as a result of their AKI.

There was a significantly greater length of stay for those patients who suffered an AKI during their inpatient stay compared to those who did not (AKI – N = 53, Mean rank 548.47, Median 7 days; No AKI – N = 728, Mean rank 379.51, Median 5 days; *p* < 0.001). There was no significant difference in 30-day mortality between those who had an AKI versus those who did not (AKI 1.9% vs No AKI 0.1%; *p* = 0.134). There was also no significant difference for 30-day readmission for those with AKI against those without (AKI 1.9% vs No AKI 2.7%; *p* = 1.00 for Fisher’s exact test).

Results of the bivariate and multivariate regression analysis regarding pre-operative variables and the outcome of AKI are shown in Table 1. Diabetes (OR 2.49; 95%CI 1.15–5.38; *p* = 0.021), CKD (OR 4.59; 95%CI 2.37–8.92; *p* < 0.001) and Male sex (OR 2.61; 95%CI 1.42–4.78; *p* = 0.002) were found to be independently predictive of AKI. Chi-squared analysis using a dummy variable containing any one of the pre-operative patient characteristics from: Diabetes, CKD, BMI ≥ 40 and Male sex was found to predict 81.1% of those who developed an AKI. The overall AKI rate for those meeting any of these three criteria was 11.2% compared to 2.5% of those without any of the three criteria (*p* < 0.001).

**Table 1:** Bivariable and multivariable logistic regression regarding potential predictors for post-operative development of acute kidney injury

	Development of acute kidney injury			
	Column 1		Column 2	
	BR OR (95% CI)	*p* value	MR OR (95% CI)	*p* value
Age	1.06 (1.03; 1.09)	< 0.001	1.03 (0.99; 1.06)	0.104
Sex = male	2.67 (1.51; 4.72)	0.001	2.61 (1.42; 0.75)	0.005
BMI	1.04 (0.99; 1.08)	0.168		
THR vs TKR	0.56 (0.30; 1.05)	0.069		
CKD	5.07 (2.66; 9.66)	< 0.001	4.59 (2.37; 8.92)	< 0.001
Ace-i/AT II	1.87 (1.01; 3.46)	0.047		
Diabetes	3.83 (1.72; 8.51)	0.001	2.49 (1.15; 5.38)	0.021
Diuretics	1.35 (0.67; 2.74)	0.404		
Pre-operative NSAIDs	0.90 (0.42; 1.92)	0.789		
CVD	2.47 (1.17; 5.21)	0.018	1.44 (0.69; 3.00)	0.336

*BMI* Body Mass Index, *THR* Total Hip Replacement, *TKR* Total Knee Replacement, *CKD* Chronic Kidney Disease, Ace-I Ace Inhibitor medication, AT II – Angiotensin II Receptor Antagonist, *NSAIDs* Non-steroidal Anti-inflammatory Drugs, *CVD* Cardiovascular Disease. *BR* Bivariate, *MR* Multivariate, *OR* Odds Ratio, *CI* Confidence Interval. Grey shading denotes statistical significance (*p* < 0.05)

Of the original 788 patients, 703 had subsequent renal function tests done after discharge (median follow-up = 943 days, range 8–1363). De novo CKD, or worsening of pre-existing CKD, occurred in 38/703 (5.4%) patients. There was no significant difference for developing CKD between those who had a post-operative AKI and those who did not (χ2 = 0.22, *p* = 0.63).

## Discussion

Since 2009, Scottish Arthroplasty Project (SAP) data [4] has demonstrated a relentless rise in the incidence of AKI in both hip and knee replacement in Scotland, to its current level of 2.2% and 1.9%, respectively. This nationally reported figure is clearly lower than the 6.8% in our current study but may be explained by the nature of national data collection which uses diagnostic coding on discharge rather than evaluation of biochemical and patient data directly [12]. This potential underreporting would appear to be confirmed by other Scottish studies which identify figures between 9 and 11%, including two national audits [5, 12–14].

We have identified three pre-operative patient factors that are independently predictive of AKI (Diabetes, CKD and Male sex). Other studies have previously examined predictive factors for the development of AKI in the setting of elective hip and knee arthroplasty. One previous smaller cohort by Kimmel et al. identified increased BMI, CKD and the use of ACEI and ARB medication as predictive of AKI through a multivariate model [15]. However, they used a significant number of variables within their analysis which likely led to model overfitting and subsequent inaccuracies in the predictive value of these characteristics. They also demonstrated a significantly greater AKI risk (14.8%) within their cohort. Abar et al. performed a large case–control study examining AKI development after joint arthroplasty. They identified higher ASA grade, CKD and large blood loss as predictive of AKI in a multivariate model [7]. Due to the matching criteria used as part of the case–control methodology, they failed to identify significant potential predictive factors such as age, sex and diabetes (the latter two of which were important independent predictors identified in this study). Ferguson et al. found both age and volume of post-operative fluids to be predictive of AKI within a similar cohort [12]. The relationship of AKI to post-operative fluid volume may provide evidence of one simple method for future targeted AKI reduction interventions.

Investigation of significant risks associated with surgical factors was not feasible within this current study, but merits further research. Previous work has identified concerns with specific prophylactic antibiotic use [16, 17], as well as large quantities of antibiotic loaded cement [18]. Peri-operative blood loss [7] and peri-operative hypotension are also potentially important adjustable factors that can increase renal hypoperfusion and worsen AKI.

Other variables such as hospital level factors [19] and mental health disorders [20] have been linked to AKI but were not specifically addressed in our study. Future analysis of large-scale registry data may provide further insight into determining the impact of these factors alongside those identified by our study and allow for external validation of our findings.

Post-operative AKI in this group was associated with a longer stay in hospital, and this concurs with the current body of recent research [5]. However, it was not associated with any significant difference in long-term outcomes (30-day mortality, 30-day readmission, development of CKD). This is contrary from the growing body of recent research which suggest that AKI is indeed associated with those aforementioned factors [1–3, 10]. It is possible an association was not seen due to the reasonably low overall number of AKI events in this study, but one other potential explanation for this discrepancy could be the mechanism of AKI as a factor in dictating the subsequent probability of developing CKD. In our patient cohort, it is felt likely that AKI development is a pre-renal phenomenon secondary to hypovolaemia as a consequence of reduced post-operative fluids [5]. Other causes that relate more directly to micro or macroscopic damage to the Kidney may be more likely to induce CKD after AKI (e.g. nephrotoxic drugs, renal ischaemia/infarction, etc.). Further investigation into the pathology behind AKI and subsequent CKD development is required to better delineate these factors.

One issue that remains unresolved however, is the progressive rise in AKI in Scotland starting in 2009 and accelerating from 2013. Whilst it is possible the rising AKI rates are related to greater awareness over time, this timeline correlates strongly with the development and roll-out of Enhanced Recovery After Surgery (ERAS) programmes throughout Scotland, with sequential audit reports indicating that between 2010 and 2012 the number of patients treated by ERAS principles in Scotland went up from 21 to 59% and then rose to 92% in 2013 [13, 14]. Though ERAS has been associated with success in reducing hospital stay and early mobilisation [21], these same reports have indicated high rates of abnormal eGFR post-operatively in those individuals who had previous normal renal function (10% by 2012 and then 11% by 2013). A review of AKI in our own patients in the ERAS pathway in 2015 indicated an overall incidence of 9.4% (5.8% when pre-operative eGFR > 60 ml/min and 33% when eGFR < 60 ml /min) [5]. One hypothesis is that AKI in these patients is in part due to the reduced post-operative fluid regimens implemented by ERAS in order to maximise early mobilisation. Such concerns have also been expressed in the use of ERAS in other surgical specialities [22]. Ongoing consideration of ways to optimise peri-operative fluid intake whilst maintaining the benefits of ERAS principles will be integral to optimising patient care in the future.

## Conclusion

In conclusion, 6.8% of patients undergoing lower limb arthroplasty developed an AKI during their inpatient stay. This was associated with a longer inpatient stay, but overall 30-day mortality and 30-day readmission rates remained the same. We identified three pre-operative patient factors (Diabetes, CKD and Male Sex) that were independently predictive of AKI, and targeted interventions towards high-risk individuals regarding peri-operative fluids may reduce the risk of AKI after lower limb arthroplasty in the future. Developing a post-operative AKI was not associated with developing subsequent CKD, but we hypothesise that this may be due to the mechanism of AKI in our patient cohort.

## Data Availability

Aggregate de-identified data available on request.

## Declaration

### Preliminary results

Preliminary results were previously published in abstract format and can be found here: https://online.boneandjoint.org.uk/doi/abs/10.1302/1358-992X.2018.18.008
